# Cultural adaptation of the workplace age-friendliness measure to German with validation in Austrian organizations

**DOI:** 10.1093/geront/gnag082

**Published:** 2026-05-06

**Authors:** Gert Lang, Raphael Eppler-Hattab

**Affiliations:** Austrian Health Promotion Fund, Austrian National Public Health Institute, Vienna, Austria; The Center for Research and Study of Aging, Faculty of Social Welfare and Health Sciences, University of Haifa, Haifa, Israel

**Keywords:** Aging workforce, Age-inclusive, Scale validation, Workplace health promotion

## Abstract

**Background and Objectives:**

The workplace age-friendliness measure is a multidimensional, four-factor instrument measuring organizational support for maintaining the employability of older workers that was developed in English and Hebrew. This study aimed to adapt it culturally for use in organizations where German is spoken and to validate its psychometric properties in the context of workplace health promotion initiatives.

**Research Design and Methods:**

The English version of the scale was cross-translated into German and piloted in a convenience sample in Austria. It was then circulated in six Austrian organizations across various industries and regions, mostly from health and education sectors, within their workplace health promotion projects, resulting in 1,725 employee responses. This sample was used to report on and validate the scale’s psychometric properties.

**Results:**

Confirmatory factor analysis demonstrated construct validity and measurement equivalence across multigroup socio-demographic variables of age, gender, and education. The results also supported the four-factor solution of the construct, with acceptable internal consistency and reliability. Correlations demonstrated the criterion (concurrent) validity of the scale with related constructs. An analysis of variance revealed meaningful differences in factor mean scores across individual and organizational characteristics.

**Discussion and Implications:**

This is the first study to develop and validate a German-language version of the workplace age-friendliness measure. As a reliable and valid instrument, it can be used to assess organizations’ practices regarding their support for an aging workforce. Further implications and limitations of using this instrument in future empirical research are discussed.

## Background and objectives

As the workforce continues to age worldwide, it is essential to develop and maintain healthy workplaces for employees of all ages. The World Health Organization emphasizes the importance of prioritizing the promotion of health and well-being in the workplace by providing safe and healthy physical and psychosocial work environments (Wolf et al., 2018). However, while maintaining healthier workplaces is gaining increasing attention to support organizational outcomes (Proper & van Oostrom, 2019), promoting health in the workplace is particularly important for older workers as they cope with occupational health-related barriers and disparities (Crawford et al., 2010; Pitt-Catsouphes et al., 2015; Poscia et al., 2016). These trends are prominently challenging for developed countries such as European countries, which are among the fastest-aging countries (Chand & Markova, 2019).

The number of workers aged 55 and older in the European Union increased from 23.8 million in 2010 to nearly 40 million in 2023, driven by higher retirement ages, longer life expectancy, and better health (Eurofound, 2025). The labor markets in German-speaking countries, including Austria, are increasingly affected by an aging workforce. The average effective retirement age in Austria is appreciably lower than the OECD average. While the standard retirement age in Austria is 65 for men and gradually increasing for women from 60 to 65 by 2033, the effective retirement age in Austria in 2024 was 62.7 for men and 61.2 for women, compared to 64.7 for men and 63.6 for women on average in OECD countries, respectively (OECD, 2025a). Moreover, in 2024, employment rates for workers aged 55–64 were 57.3%, compared to 62.3% on average in OECD countries, with employment in the 60–64 age group remaining particularly low (48.1% for men and 20.0% for women) (OECD, 2025b). These trends reinforce the need to promote health in organizations to support the retention of older workers in the labor market.

Against this backdrop, workplace health promotion (WHP; i.e., combined efforts of employers, employees, and society to improve the health and well-being of people at work) (ENWHP, 2007) in countries with an increasingly aging workforce, including Austria, needs to focus more strongly on addressing the health issues of an aging workforce in general and of older workers in particular. An effective way to advance this goal is to incorporate quantitative measurement to aid organizations in managing and maintaining the health of their aging employees. While there are a limited number of scales for measuring WHP (e.g., Yener et al., 2024), scales covering the intersection of aging and health in workplaces, such as the health management pillar of the Later Life Workplace Index (LLWI) (Wilckens et al., 2021), are rather scarce. We, thus, chose to adapt the Workplace Age-Friendliness Measure (WAFM) (Eppler-Hattab et al., 2020a, 2020b) for the current study to support health promotion in organizations in German-speaking countries. We chose this instrument since, unlike other measures related to aging and work (e.g., LLWI), this measure was developed from an organizational perspective that specifically targets issues of organizational culture and climate related to older workers by implementing it within identifiable organizations that allow for organizational-level analysis. It is also a relatively short instrument and can be easily delivered in organizations. Notably, although the WAFM and the LLWI share similar domains, the original LLWI contains 80 items, and a shorter 29-item version has been published recently (Finsel et al., 2025), and thus, was not available at the time of our study. Finally, it contains several health-related dimensions such as employee health and well-being, employee development, and employment flexibility, which are essential components of occupational health promotion in organizations (Grosch & Scholl, 2020).

The purpose of this study is, therefore, to present the cultural adaptation of the WAFM and the psychometric validation of its German version using a sample of 1,725 employees from six Austrian organizations. We empirically examine the reliability and validity of the developed measure (Eppler-Hattab et al., 2020a) for further use in German-speaking populations, primarily in the context of WHP. By adopting univariate, bivariate, and multivariate analytical approaches as well as using different structural equation models (SEM), we demonstrate the robustness of the German scale across the various characteristics of the sample. For this purpose, the following research hypotheses were deduced:

1. Measurement hypothesis: We hypothesized that the German-language version of the WAFM has the same dimensional structure as the English-language version, with acceptable factorial reliability and validity.
2. Measurement equivalence hypothesis: We hypothesized that the WAFM dimensions are measured in the same way across different sociodemographic groups and that the observed differences were true differences in the dimensions rather than measurement bias.
3. Criterion validity hypothesis: We hypothesized that the German WAFM is related to other scales measuring relevant organizational outcomes.

We conclude by discussing the implications of our research results and their potential theoretical and practical contributions.

## Theoretical background

### Workplace health promotion

The promotion of healthy workplaces is intrinsically drawn from theories of organizational change (Batras et al., 2016). The primary foundation for our measurement research concerns Lewin’s theories of organizational change (Lewin, 1997), along with subsequent organizational behavior understandings of change management (Bartunek & Woodman, 2015). Together with organizational culture theory (Schein, 2010; Schneider et al., 2013), this view underpins the organizational dynamics essential to the development and success of WHP. Specifically, according to the Luxembourg Declaration on WHP in the European Union (ENWHP, 2007), the process of creating healthy workplaces is based on a comprehensive design and analysis of individual and organizational factors at different health and work-related levels. The WHP approach has great potential due to its focus on the workplace as an intervention setting and can reach a large working population (Shain & Kramer, 2004). Systematic reviews confirm the effectiveness of WHP in fostering a culture of health and well-being and improving work capacity and productivity, especially when comprehensive (i.e., multimodal, holistic) programs are implemented (Goldgruber & Ahrens, 2010; Poscia et al., 2016; Tam & Yeung, 2018). Moreover, the Austrian Network for WHP has developed a validated management system for quality assurance, whose quality evaluation procedure in WHP measures 15 quality criteria and allows WHP to be assessed in relation to quality and success (Lang & Jiménez, 2024). In line with quality concepts for injury prevention and health promotion (Donabedian, 1988), these quality criteria distinguish between the dimensions of the structure, process, and outcome of WHP. The structure of WHP refers to the relevant framework conditions and prerequisites, such as organizational culture, available resources, and contact persons. The process takes the way in which WHP is provided into account, including target group orientation (in our case, aging employees), process planning, diagnostic methods, and the implementation of measures. Finally, the outcome deals with monitoring and evaluating, for instance, the effectiveness, employee satisfaction, and sustainability of a WHP in a given organization (Lang & Jiménez, 2024).

The success of implementing WHP programs is largely dependent on organizational behavior factors such as the organization’s culture and its capacity to change. Considering this perspective is fundamental to WHP, as it provides a conceptual basis for designing, implementing, and evaluating effective WHP programs. There are several key reasons for this consideration. First, WHP interventions rely on behavior change at multiple levels, namely individual, interpersonal, and cultural. A review of behavior change theories (Koulouvari et al., 2025) showed that workplace research-based interventions of health promotion are more effective as they target key mechanisms that influence employee behavior and working conditions, emphasizing the importance of multilevel approaches to health promotion interventions. Second, organizational and psychosocial mechanisms that may influence health and well-being form the basis for interventions targeting, including psychosocial risks, health promotion climate, and leadership commitment (Tetrick & Peiró, 2016). Third, intervention research has shown that organizational factors (e.g., leadership, resources, climate, structure, and norms) are critical determinants of successful WHP adoption. In this sense, organizational resources provide the framework for an effective WHP, thus helping to systematically analyze such determinants (Weiner et al., 2009). Finally, this conceptual basis may assist in better understanding organizational change processes that are central to WHP implementation, thus providing insights into internal dynamics, readiness for change, leadership roles, and contextual factors that influence the extent to which WHP interventions are culturally and normatively embedded (Batras et al., 2016).

WHP can, therefore, play a substantive role for organizations when dealing with contemporary challenges of working life, particularly with regard to the demographic change of an aging workforce. This is likely because the average work ability of employees may decline with age if targeted measures to promote and maintain health and well-being at the workplace are not implemented (for a review, see Cadiz et al., 2019). Positive effects can, therefore, be achieved with individual-directed WHP measures on a behavioral level (Poscia et al., 2016). However, sustainable gains may be better achieved when both context-related and situation-related aspects on an organizational level are identified and implemented in corresponding environment-directed WHP measures. For example, such implementation may aid in improving the physical-social working environment, organizational norms, and leadership behavior (Cadiz et al., 2019; Ilmarinen, 2002).

### Workplace age-friendliness

Organizational culture and climate can have a significant impact on the extent to which employees can age successfully in the workplace (Eppler-Hattab et al., 2020b; Zacher & Yang, 2016). Age-related organizational culture and climate refer to shared beliefs and perceptions about age and aging at work, which influence how older workers thrive and survive in organizations over time while impacting workplace health outcomes such as age discrimination and maintaining motivation and performance (Rudolph et al., 2018; Zacher & Froidevaux, 2021). Research on age-related organizational culture and climate and their role in addressing organizational challenges associated with an age-diverse and older workforce is growing rapidly as the workforce continues to age (e.g., Appannah & Biggs, 2015; Beier et al., 2022; Eppler-Hattab et al., 2024). In this field, there is a general agreement that promoting age-supportive work environments is a necessary component to prolong working lives (Boehm et al., 2021; Eppler-Hattab et al., 2020b).

However, unlike age-inclusive approaches to supporting an age-diverse workforce (Boehm et al., 2021; Truxillo et al., 2015), Workplace Age-Friendliness (WAF) refers more specifically to older workers within aging workforces. It is conceptualized as the extent to which an organization maintains the employability of its older workers by fostering an organizational culture in which they are accepted and treated according to their competencies and needs (Eppler-Hattab et al., 2020b). This approach, intertwined with age-diverse, reciprocal social exchange processes (Kitz et al., 2025), suggests that promoting an age-friendly organizational culture can convey a positive message to employees of different age groups, thereby fostering continuous healthy employment across individuals’ working lifespans (Dietz & Fasbender, 2022).

Constructs of organizational adaptation to working in later life tend to be multidimensional given the variety of physiological and psychological aspects as well as organizational mechanisms associated with such organizational approaches (Chen & Gardiner, 2019; Wilckens et al., 2021). Appropriately, an “age-friendly workplace” theoretically consists of an age-friendly core culture dimension reflecting age-related organizational values, driving four organizational climate dimensions: lifelong employee development and learning, psychological and physical health and well-being, job (re)design, and employment flexibility (Eppler-Hattab et al., 2020b). In a series of studies that developed this multidimensional construct by using exploratory and confirmatory factor analytic techniques, the WAFM was validated as consisting of four dimensions: (1) core culture (age-friendly organizational values such as fairness and equitability in recruitment, promotion, and retirement, as well as maintaining an age-diversity climate), (2) employee development (e.g., through upskilling and reskilling of employees), (3) employee health and well-being, and (4) employment flexibility (Eppler-Hattab et al., 2020a, 2024).

### Integrating health promotion and age-friendliness in the workplace

Healthy workplaces and age-friendliness in the workplace can be viewed as interrelated concepts (Grosch & Scholl, 2020; Truxillo et al., 2015). Given the increasing rate of changes in occupational health outcomes that occur with aging, an occupational health perspective on workplace design can optimize the safety, health, and well-being of aging workers. An age-friendly environment in this context is designed to enable the continued optimal employment of older and aging workers, including through changes in the work environment and in human resources policies, expanding flexible working practices, encouraging lifelong workplace learning, and adopting a holistic approach to employee health in the workplace (Appannah & Biggs, 2015; Poscia et al., 2016).

For example, Truxillo et al. (2015) proposed a health-oriented approach to age-friendly management practices in organizations, categorized as primary, secondary, and tertiary workplace health interventions. Based on a review of 59 publications, aging-related physical and psychological changes may be mitigated by health promotion programs such as increased physical activity, intellectual activity, and healthy nutrition (Crawford et al., 2010). A further review by Poscia et al. (2016) stressed the need for well-designed WHP programs to advance a more systematic investigation of their effectiveness and cost benefits for organizations employing an age-diverse workforce. These health-driven approaches are reflected in the concept of age-friendliness in the workplace (Eppler-Hattab et al., 2020b) and its organizational measure (Eppler-Hattab et al., 2020a) and can, thus, help advance these goals.

**Figure 1:**
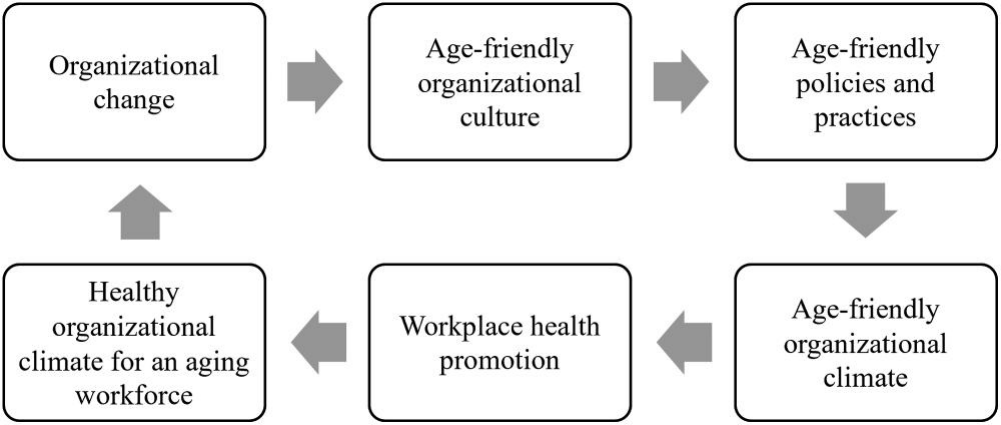
Flowchart of working theories related to cultural adaptation of the workplace age-friendliness measure (WAFM) for workplace health promotion (WHP).

To conclude this section, we outline a flowchart summarizing the working theories underlying the cultural adaptation of the WAFM for WHP initiatives (Figure 1). This flowchart is based on the premise that organizational change management is necessary to facilitate the process of creating a healthy organizational climate for an aging workforce (Bartunek & Woodman, 2015; Batras et al., 2016). Based on Eppler-Hattab et al.’s (2020b) conceptualization of WAF, coupled with Ostroff et al.’s (2013) theory of organizational culture and climate, an age-friendly organizational culture can drive supportive age-friendly policies and practices, which in turn can create shared perceptions emerging into an age-friendly organizational climate. This process can further support an age-related WHP (Goldgruber & Ahrens, 2010; Shain & Kramer, 2004), which ultimately, supported by ongoing measurement, as we suggest, can create and maintain a healthier organizational climate for an aging workforce. Continuous use of the measure can provide organizations with an assessment of the progress of efforts to maintain and improve age-related workplace health while gaining feedback on further organizational change needed for WHP.

## Research design and methods

### Translation and pretest validation

Based on guidelines for a cross-cultural adaptation of self-report measures (Beaton et al., 2000), six Austrian health and translation experts adapted the original WAFM from English to German, and then, translated it back to English. On this basis, this panel of experts compiled the pretest version. This version was tested in a pilot study by drawing a convenience sample of 168 Austrian employees using the snowball method in an online survey. To confirm the German version, analyses attested to the quality of the translation. For example, the translated items achieved acceptable distribution characteristics, and the factorial structure of the original study was also confirmed in this pretest validation. In line with the validation procedure of Eppler-Hattab et al. (2020a), additional confirmation was achieved by assessing the criterion validity of the German-language instrument with related constructs, including scales of perceived organizational support, climate for diversity, workplace age stereotypes, and intergroup (inter-age) contact. In the final version, some individual improvements were made to the introduction (e.g., an additional explanation as to which types of organizations “my organization” might include) as well as to the wording (e.g., using the German term for “well-being” instead of “wellness”) and length of specific items based on qualitative feedback from respondents in the pilot study.

### Instrumentation

In this study, the 24 items of the final German translation of the WAFM were included in the standardized combined questionnaire on work and health (German: *Kombinationsfragebogen Arbeit und Gesundheit*; hereafter: KombiAG). This questionnaire was developed in Austria in the context of WHP projects and is applied to identify mental workloads for workplace evaluation (Jiménez & Dunkl, 2017) in accordance with the Austrian Worker Protection Act and the Austrian Employee Protection Act. The KombiAG questionnaire measures several health-related aspects with validated multiple-item scales, including work tasks and activities (8 items; e.g., “I had to deal with unpleasant situations at work like conflicts, illness, accidents”), social and organizational climate (8 items; e.g., “I was able to rely on my direct superior at work”), manager support (5 items; e.g., “My manager gives me constructive feedback”), and colleague support (4 items; e.g., “I can rely on my colleagues”), corporate culture (3 items; e.g., “The well-being and health of the employees are of great importance in this company”), appreciation/gratification (4 items; e.g., “Appreciation is demonstrated in this company”), organizational dissatisfaction (7 items; e.g., “How satisfied are you with the organization and leadership?”), and job dissatisfaction (5 items; e.g., “How satisfied are you with the type and content of your work?”). Each scale had a five-point answer format (e.g., from 1 = never to 5 = always, or from 1 = very satisfied to 5 = very dissatisfied).

### Sample

Data were collected during WHP projects with a focus on transition management in six Austrian organizations from various industries, sectors, and regions. The organizational surveys were implemented for WHP needs analysis between April and October 2024. The target population was all employees at the organizations, based on the premise that all employees, regardless of their age, experience the culture of their organization with regard to age (see Eppler-Hattab et al., 2020a). They were asked to respond to a voluntary and anonymous online questionnaire respecting national data protection regulations based on a contract between the Austrian National Public Health Institute and each participating organization. Participants gave informed consent before entering into the study. They were not compensated for their participation.

In total, a response rate of 52.1% was achieved on average, with 1,725 employees answering the German WAFM items. The total sample consisted of 3.7% respondents aged up to 24, 21.9% aged 25–34, 28.8% aged 35–44, 27.7% aged 45–54, and 16.2% aged 55 or over (1.8% of the respondents did not specify their age). Based on personnel statistics of the organizations, 17.2% of all employees were aged 55 or older on average. Because the study was primarily conducted in education and healthcare sectors, with a majority of female employees, the average proportion of women was relatively high (81.6%). More detailed sample descriptions by organizations are presented in Table 1.

**Table 1:** Sample descriptives of the study sample.

		Organization						
Variable	Description	A	B	C	D	E	F	Total
**Industry**	Economic classification^a^	Education	Education	Health	Health	Health	Other	
**Type**	(Non-)profit or public	Nonprofit	Public	Nonprofit	Profit	Nonprofit	Nonprofit	
**Region**	By federal state^b^	Eastern Austria	Eastern Austria	Eastern Austria	Southern Austria	Western Austria	Austria-wide	
**Demography**	% of employees 55+^c^	15.6%	15.0%^d^	12.8%	12.0%	17.9%	23.3%	17.2%
**Sample size**	*n*	68	86	268	331	748	224	1,725
**Response**	Response rate	27.2%	29.0%	60.2%	73.4%	56.5%	41.2%	52.1%
**Gender**	% female	75.0%	64.0%	69.4%	95.2%	87.4%	65.2%	81.6%
**Age**	% 55+	14.7%	15.1%	12.3%	10.9%	18.9%	20.5%	16.2%
**Education^e^**	% (applied) university	57.4%	75.6%	n.a.	22.1%	13.1%	30.4%	19.9%
**Position^f^**	% executive, manager	79.4%	n.a.	11.6%	46.5%	49.6%	72.8%	44.8%
**Employment^g^**	% full-time employed	57.4%	80.2%	18.3%	23.0%	30.5%	68.3%	35.6%
**Duration^h^**	% 10+ years in the organization	32.4%	46.5%	31.0%	24.8%	22.1%	50.4%	29.3%

*Note*. n.a. = not available.

a An Austrian classification of economic activities in the European Union ÖNACE code, NACE “Nomenclature statistique des activités économiques dans la Communauté européenne”: Education and training, health and social services, or provision of other services.

b Eastern Austria (incl. Burgenland, Lower Austria, Vienna), western Austria (Upper Austria, Salzburg, Tyrol, Vorarlberg), southern Austria (Carinthia, Styria).

c Personnel statistics of the organizations.

d Estimated based on the number of employees aged 50+.

e In response to the question: “What is your highest level of education?”

f “What is your current professional position?”

g “To what extent are they employed?”

h “How long have you been working for the company?”

### Analysis

Following Hinkin’s (2005) principles for scale development and the recommendations by Kline (2011) for using SEM, the appropriateness for multivariate analysis was checked by univariate and bivariate analyses: Because SEM relies on the analysis of variance-covariance matrices, items were analyzed descriptively using item means (*m*) and standard deviations (*s*^2^) with the expectation that variables would be dispersed around the mean with unimodal distributions (*s*^4^ < |7.0|) and no items with particularly high skewness (*s*^3^ < |2.0|) (Byrne, 2012). In addition, correlation patterns were used to check whether items of a dimension converge and whether they discriminate with respect to items from other dimensions.

To test the psychometric properties of the German WAFM, several steps were implemented. First, the structure and accuracy of the WAFM were investigated with Confirmatory Factor Analysis (CFA) to examine its factorial reliability and validity using the statistical modeling program Mplus 8. This approach was used as exploratory factor analysis had been performed in the development of the English-language instrument. However, the current study aimed to confirm the tested factor structure with the German-language instrument, and therefore, CFA was implemented. Models were executed with the maximum likelihood estimator, and in the case of skewed items, the maximum likelihood estimator with robust standard errors was used. Following the recommendations in the literature (Bagozzi, 1984; Fornell & Larcker, 1981; Nunnally & Bernstein, 1994), construct validity and reliability were checked using the following criteria (and accepted thresholds): the validity (*λ* >0.50) and reliability (*R*^2^ > 0.40) of indicators, internal consistency reliability (Cronbach’s alpha [*α*] and McDonald’s Omega [*ω*] >.70), and average variance extracted (AVE >0.50). Absolute and comparative fit indices served to assess the goodness of fit of the overall model, using the chi-square test (χ^2^/degrees of freedom [*df*] <3.0), root mean standard error of approximation (RMSEA <0.08), comparative fit index (CFI >0.90), standardized root mean square residual (SRMR <0.08) for an acceptable fit, and CFI >.95, RMSEA, and SRMR <.050 for a good fit (Bollen, 1989; Browne & Cudeck, 1993; Hu & Bentler, 1999; Kline, 2011). In case the CFA assumptions turned out to be too restrictive, the residual matrix and modification indices were consulted, and only theoretically feasible modifications and plausible measurement error co-variances were allowed, as previously discussed in the literature (e.g., Cole et al., 2007). The level of statistical significance was set to *p* < .05.

Second, we tested whether the German WAFM measures have an interpretable metric and measurement equivalence using multigroup CFA across age, gender, and education of respondents. These represent key demographic subgroups in organizations and are relevant variables for WAF (Eppler-Hattab et al., 2024). As such, they can potentially influence language interpretation, cultural norms, and work-life experiences. Testing measurement equivalence across these subgroups ensures that the WAFM was valid for diverse populations and was not biased by subgroup differences. For testing measurement equivalence, configural (no restrictions at all), metric (equal slopes), and scalar measurement equivalence (equal slopes and intercepts) models were compared against each other, where a nonsignificant Satorra–Bentler χ^2^ difference test (*p* > .050) and a difference of CFI <0.01 between two nested models indicated the presence of measurement equivalence (Cheung & Rensvold, 2002). If the assumptions were too restrictive, partial measurement equivalence was also considered (Cloos & Flake, 2022), for example, by releasing constraints on individual items (slopes and intercepts).

Third, criterion validity was evaluated using correlated factor models with the aforementioned KombiAG scales. The association was assessed using Pearson correlation coefficients, and its strength of association was evaluated by Cohen’s (1992) classification of effect sizes (i.e., small effect *r* = 0.10, medium *r* = 0.30, large *r* = 0.50).

Finally, we present a description of the properties using factor scores for each WAF dimension, including factor means and standard deviations on the theoretical scale range between a minimum value of 1 and a maximum value of 7, and in relation to statistically significant differences between relevant groups. These included socio-demographic variables of gender (male versus female), age groups (younger employees aged up to 44 years versus older employees aged 45 years or older based on the sample age distribution), and education level (lower versus higher education), as well as organizational variables such as organization type (public, profit, nonprofit), industry (education, health, other), and organizational size (50–250, 251–750, more than 750 employees).

## Results

### Univariate and bivariate item analyses

Table 2 shows the descriptive statistics of the 24 WAFM items using a 7-point bipolar answer format (1 = absolutely disagree to 7 = absolutely agree), grouped by the four dimensions of the WAFM. On average, the items varied considerably around the total item mean of *m*_total_ = 5.7 ($s_{\text{total}}^{2}$ = 1.6). Item means for core culture (*m *= 6.1) and development (*m *= 5.7) were on average higher than those for well-being (*m *= 5.4) and flexibility (*m *= 4.9). In contrast, the standard deviations were the highest for flexibility items (*s*^2^ = 2.0) and lower for well-being (*s*^2^ = 1.8), development (*s*^2^ = 1.6), and core culture items (*s*^2^ = 1.4). The items were mostly slightly skewed (*s*³ = −1.4, *s*³_min_ = −2.1, *s*³_max_ = −0.3) and had predominantly unimodal distribution (*s*^4^ = 1.5, $s_{\text{min}}^{4}$= −1.3, $s_{\text{max}}^{4}$ = 4.3). The correlation coefficients between items within a WAF dimension were higher (convergent validity) than items between WAF dimensions (discriminant validity), indicating construct validity. Core culture items were intercorrelated from *r* = 0.53 to 0.80, development items from *r* = 0.57 to 0.82, well-being items from *r* = 0.74 to 0.89, and flexibility items from *r* = 0.65 to 0.82 (*p* < .001). Hence, the underlying data were suitable for conducting multivariate analysis. To account for item skewness, the maximum likelihood estimator with robust standard errors was used.

**Table 2:** Descriptive statistics of the German WAFM items.

Item	German/English wording^a^	*m*	*s*²	*s*³	*s* ^4^
	**Kernkultur/core culture**				
**c1**	Meine Organisation behandelt ältere Beschäftigte fair und gleichberechtigt./My organization treats older workers fairly and equally.	6.1	1.4	−1.9	3.1
**c2**	In meiner Organisation gibt es keine Altersdiskriminierung in Abläufen wie bei Bewerbung, Beförderung und Kündigung./In my organization, there is no age discrimination in processes such as recruitment, promotion, and dismissal.	6.1	1.6	−2.0	3.0
**c3**	Der Wunsch meiner Organisation, Beschäftigte aller Altersstufen (einschließlich älterer Beschäftigter) einstellen und halten zu wollen, wird von ihren Führungskräften umgesetzt./Managers in my organization are a personal example of the wish to recruit and retain workers of all ages, including older workers.	6.0	1.4	−1.8	3.0
**c4**	In meiner Organisation gibt es eine positive Atmosphäre gegenüber der Beschäftigung älterer Mitarbeiter:innen./In my organization, there is a positive atmosphere toward the employment of older workers.	6.2	1.3	−2.0	4.1
**c5**	Meine Organisation unterstützt eine Vielfalt von Altersgruppen in ihrer Belegschaft./My organization promotes multi-age diversity in the organizational workforce.	6.2	1.3	−2.0	4.2
**c6**	Meine Organisation stellt sicher, dass ältere Beschäftigte in einem nicht geringeren Ausmaß als andere Beschäftigte anerkannt und respektiert werden./My organization makes sure that older workers are recognized and respected no less than other workers.	6.2	1.3	−2.1	4.3
**c7**	Meine Organisation zeigt Verantwortung für ältere Beschäftigte, die schon lange einen Beitrag für sie leisten./My organization shows responsibility for older workers who have long contributed to the organization.	6.1	1.4	−1.8	2.6
**c8**	Ältere Beschäftigte in meiner Organisation stehen bei Entlassungen in Zeiten betrieblicher Veränderung oder Stellenabbau nicht an erster Stelle./Older workers in my organization are not the first priority for dismissal during organizational change or downsizing.	6.0	1.6	−1.8	2.3
**c9**	Ältere Beschäftigte in meiner Organisation werden nicht unter Druck gesetzt, ihren Platz zur Verfügung zu stellen und frühzeitig aus dem Erwerbsleben auszuscheiden./Older workers in my organization are not pressured to vacate their place and retire early.	6.1	1.6	−2.0	3.1
	**Entwicklung/development**				
**d10**	Meine Organisation erlaubt älteren Beschäftigten, deren Wissen und Kompetenzen als Teil ihres Berufs zu aktualisieren und zu verbessern./My organization allows older workers to update and upgrade their knowledge and skills as part of their job.	6.2	1.2	−1.9	3.7
**d11**	In meiner Organisation werden ältere Beschäftigte dazu ermutigt, neue Kompetenzen zu erwerben, die für Veränderungen in ihrem Berufsfeld geeignet sind./In my organization, older workers are encouraged to acquire more new skills appropriate for changes in their professional field.	5.9	1.5	−1.5	1.8
**d12**	In meiner Organisation werden ältere Beschäftigte dazu ermutigt, für andere Arbeitnehmer: innen als Mentorinnen bzw. Mentoren zu fungieren./In my organization older workers are encouraged to serve as mentors for other employees.	5.1	2.0	−0.8	−0.6
**d13**	Meine Organisation ermöglicht älteren Beschäftigten sich während ihres Erwerbslebens kontinuierlich weiterzuentwickeln./My organization allows older workers to continue to develop throughout their working lives.	6.0	1.3	−1.6	2.3
**d14**	In meiner Organisation werden ältere Beschäftigte dazu ermutigt, Veränderungen in ihrem Beruf zu initiieren, je nach den Bedarfen der Organisation./In my organization, older workers are encouraged to initiate changes in their jobs, in line with the needs of the organization.	5.4	1.7	−1.0	0.1
**d15**	Meine Organisation weiß, wie sie von dem Gesamtwissen, den Kompetenzen und Fähigkeiten älterer Beschäftigter profitiert./My organization knows how to benefit from the total knowledge, skills, and abilities of older workers.	5.7	1.7	−1.3	0.9
	**Gesundheit und Wohlbefinden/health and well-being**				
**w16**	Meine Organisation fördert und kümmert sich um die Gesundheit und das Wohlbefinden älterer Beschäftigter./My organization takes care and acts to promote the health and well-being of older workers.	5.6	1.6	−1.1	0.4
**w17**	Meine Organisation ermutigt ältere Beschäftigte, an gesundheitsfördernden Aktivitäten teilzunehmen./My organization encourages older workers to participate in health promotion activities.	5.5	1.7	−1.1	0.2
**w18**	Meine Organisation arbeitet daran das Bewusstsein und die Einstellung zur Weiterbeschäftigung im fortgeschrittenen Alter zu ändern./My organization works to raise awareness and change attitudes toward continuing work at older ages.	5.4	1.8	−1.1	0.1
**w19**	In meiner Organisation wird älteren Beschäftigten, wenn nötig, eine berufliche Veränderung angeboten, damit ihren Fähigkeiten besser entsprochen wird./In my organization, older workers are offered job changes, if necessary, to better fit their abilities.	5.0	2.0	−0.7	−0.8
**w20**	Meine Organisation gestaltet die Arbeit so, dass ältere Beschäftigte in ihrer optimalen Funktion im Betrieb bleiben können./My organization organizes the work so that older workers remain in the organization in optimal functioning.	5.4	1.8	−1.0	−0.2
**w21**	Wenn erforderlich unterstützt meine Organisation dabei, physische oder psychische Anforderungen zu reduzieren oder den Fähigkeiten und Bedürfnissen älterer Beschäftigter anzupassen./When required, my organization helps to reduce or adapt physical or psychological efforts to the abilities and needs of older workers.	5.3	1.9	−0.9	−0.3
	**Flexibilität/flexibility**				
**f22**	An meinem Arbeitsplatz wird älteren Beschäftigten die Flexibilität zugestanden, den Umfang der geleisteten Arbeitsstunden zu wählen./In my workplace, older workers are given flexibility in choosing the range of hours worked.	5.6	1.8	−1.2	0.5
**f23**	An meinem Arbeitsplatz wird älteren Beschäftigten die Flexibilität zugestanden, den Umfang des Arbeitsbereichs zu wählen./In my workplace, older workers are given flexibility in choosing the scope of the position.	4.8	2.0	−0.6	−1.0
**f24**	An meinem Arbeitsplatz wird älteren Beschäftigten die Flexibilität zugestanden, den Arbeitsort zu wählen./In my workplace, older workers are given flexibility in choosing the job location.	4.5	2.2	−0.3	−1.3

*Note*. *N* = 1,725; m mean, *s*^2^ standard deviation, *s*³ skewness, *s*^4^ kurtosis; responses ranged from 1 (absolutely disagree) to 7 (strongly agree).

a To facilitate understanding, both the German-language and the English-language items are presented.

### Dimensionality of the German WAFM

As expected, the baseline CFA model that measured WAF as a single factor revealed no fit to the data (χ^2^ = 3,053.03, *df* = 252, RMSEA = 0.08, CFI = 0.77, SRMR = 0.08). Instead, the model with four factors that differentiated between core culture, development, health and well-being, and flexibility, as WAF dimensions, resulted in an acceptable fit (χ^2^ = 1,075.05, *df* = 246, RMSEA = 0.04, CFI = 0.93, SRMR = 0.05). Modification indices suggested adding error covariances between individual items, and the four specified items (i.e., between items c8–9, d10–11, d10–13, w16–17) resulted in a good model fit (χ^2^ = 877.79, *df* = 242, RMSEA = 0.04, CFI = 0.95, SRMR = 0.05). In contrast, the alternative model with five factors (core culture, development, sustainment, modification, and flexibility, as WAF dimensions) (Eppler-Hattab et al., 2020b) revealed an unacceptable fit (χ^2^ = 1,707.59, *df* = 242, RMSEA = 0.06, CFI = 0.88, SRMR = 0.08). Specifying the identical four error covariances as mentioned above yielded a just acceptable fit (χ^2^ = 1,373.88, *df* = 238, RMSEA = 0.05, CFI = 0.91, SRMR = 0.08). Therefore, the four-factor model (with four error covariances) was accepted.

**Figure 2:**
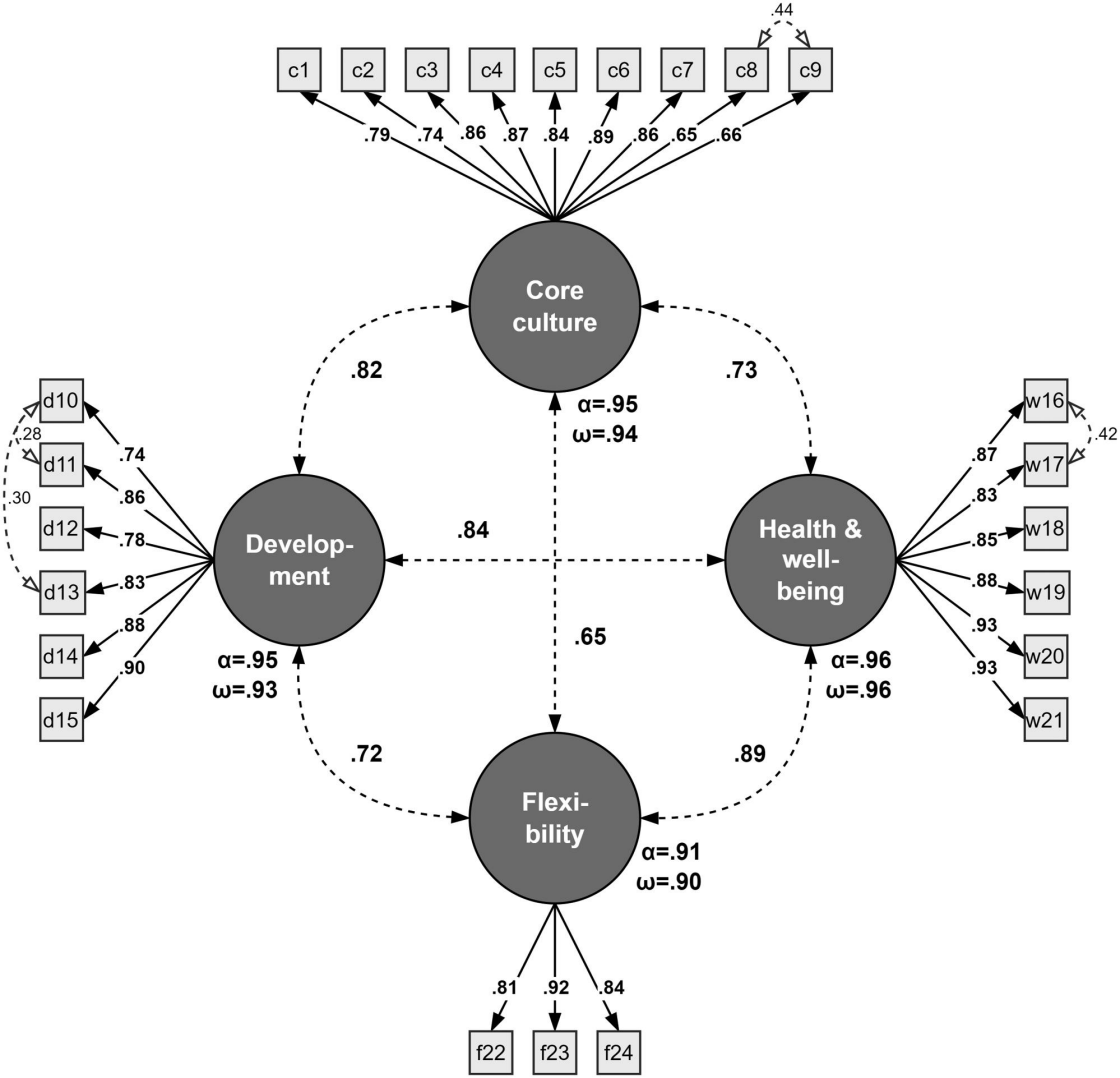
Standardized CFA results with four dimensions of the German WAFM.

The measures were characterized by high factor loadings (core culture items: λ = 0.65 to 0.89, development items: λ = 0.74 to 0.90, health and well-being items: λ = 0.83 to 0.93, flexibility items: λ = 0.81 to 0.92) and high explained indicator variance (core culture: *R*^2^ ≥0.42, development: *R*^2^ ≥0.55, health and well-being: *R*^2^ ≥0.68, flexibility: *R*^2^ ≥0.65). The average variance extracted per dimension was 0.62 for core culture, 0.71 for development, 0.78 for health and well-being, and 0.74 for flexibility. Cronbach’s Alpha and McDonald’s Omega values indicated that all dimensions were measured with high precision (core culture: *α* = 0.95/*ω* = 0.94, development: *α* = 0.95/*ω* = 0.93, health and well-being: *α* = 0.96/*ω* = 0.96, flexibility: *α* = 0.91/*ω* = 0.90). Structurally, the dimensions correlated substantially from *r* = 0.72 (between development and flexibility) to *r* = 0.89 (between health and well-being and flexibility, all *p* < .001), indicating a common source of WAF. The results are shown in Figure 2.

### Equivalence tests of the German WAFM

To test invariance, the model was examined at increasingly restrictive levels of measurement equivalence. The multigroup CFA showed that strong measurement equivalence was obtained for gender groups (male versus female) as both the restriction of equal slopes [metric model: Δ χ^2^ (20) = 22.15, *p* = .333; ΔCFI = −.001] and equal intercepts [scalar model: Δ χ^2^ (20) = 24.56, *p* = .219; ΔCFI = −.001] did not significantly worsen model fit (χ^2^ = 1,349.57, *df* = 524, RMSEA = 0.04, CFI = 0.94, SRMR = 0.06). There was also partial strong measurement equivalence for age, as there was no significant deterioration of model fit (χ^2^ = 1,239.68, *df* = 521, RMSEA = 0.04, CFI = 0.94, SRMR = 0.06) after releasing two slopes [metric model: Δ χ^2^ (18) = 28.32, *p* =.057; ΔCFI = −.001] and one intercept parameter [scalar model: χ^2^ (19) = 27.74, *p* = .089; ΔCFI = −.001] across age groups (age <45 versus age 45 and over). In addition, there was partial strong measurement equivalence for education groups (no qualification versus general qualification for university entrance) after releasing two slopes [metric model: Δ χ^2^ (18) = 27.77, *p* = .066; scalar model: Δ χ^2^ (20) = 29.52, *p* = .078; both: ΔCFI = −.001], with acceptable model fit (χ^2^ = 1,146.73, *df* = 522, RMSEA = 0.04, CFI = 0.94, SRMR = 0.06).

These results indicate that the WAF measures were consistent across relevant socio-demographic groups. This means that the WAFM is consistently interpreted and functions in the same way across gender, age, and education groups. This result ensures that differences in scores between groups reflect true differences in the dimensions rather than differences in how the instrument is understood or interpreted by the groups.

### Criterion validity of WAF dimensions

To test criterion validity, the dimensions of the WAFM were correlated against the KombiAG scales. We expected to find moderate positive correlations between KombiAG scales and the WAFM except for organizational dissatisfaction and job dissatisfaction, for which we expected to find negative correlations. Table 3 reports zero-order correlations of the study variables. The results revealed that the relationships between the four dimensions and the KombiAG scales are largely consistent with our expectations: Scores correlated strongly with those on scales measuring organizational aspects such as positively experienced corporate culture (*r* = 0.55 to 0.70) and appreciation/gratification on the part of managers, colleagues, and the organization (*r* = 0.57 to 0.70), the level of organizational dissatisfaction (*r* = −0.50 to –0.60), and a favorable social/organizational climate (*r* = 0.49 to 0.54). They were moderately to strongly correlated for increasing levels of job dissatisfaction (*r* = −0.42 to –0.51) and manager support (*r* = 0.43 to –0.50) and moderately correlated for colleague support (*r* = 0.36 to 0.41) and the degree of demanding work tasks and activities (*r* = 0.26 to 0.36; *p* < .001 for all).

**Table 3:** Zero-order correlations of WAF dimensions.

	Pearson correlation (*r*)				Reliability	
Variable	Core culture	Development	Health & well-being	Flexibility	*α*	*ω*
**Company/corporate culture**	+0.55 ***	+0.60 ***	+0.70 ***	+0.63 ***	0.87	0.88
**Appreciation/gratification**	+0.57 ***	+0.64 ***	+0.70 ***	+0.65 ***	0.77	0.78
**Organizational dissatisfaction**	–0.50 ***	–0.59 ***	–0.56 ***	–0.57 ***	0.87	0.87
**Social and organizational climate**	+0.49 ***	+0.52 ***	+0.54 ***	+0.51 ***	0.79	0.81
**Job dissatisfaction**	–0.42 ***	–0.49 ***	–0.51 ***	–0.46 ***	0.78	0.78
**Manager support (direct supervisor)**	+0.43 ***	+0.50 ***	+0.50 ***	+0.45 ***	0.93	0.93
**Colleague support**	+0.36 ***	+0.39 ***	+0.41 ***	+0.38 ***	0.82	0.82
**Work tasks and activities**	+0.26 ***	+0.28 ***	+0.36 ***	+0.36 ***	0.74	0.73

*Note*. *N* = 1,725; *α* Cronbach’s alpha, *ω* McDonald’s omega coefficient.

*** *p* ≤ .001.

### Properties of the WAFM scales

The factor scores of the four scales of the WAFM varied in terms of their means and standard deviations, from *m *= 6.2 (*s*^2^ = 0.8) for the development dimension and *m *= 5.5 (*s*^2^ = 1.3) for the health and well-being dimension. Based on an ANOVA, Table 4 shows significant mean differences in the scales by individual and organizational variables. As a measure of the effect size, *R*^2^ indicates the proportion of the total variance of the WAF scales explained by the differences between groups. Indicatively, the effects for the six different organizations (A–F) were between *R*^2^ = 0.06 to 0.09 across all WAF scales, while for groups of industries (education and training, health and social services, and others) the effects were between *R*^2^ = 0.05 to 0.06, and for organization types the effects were between *R*^2^ = 0.03 to 0.04. In contrast, the effect sizes for gender, age, and education groups were lower, at *R*^2^ = 0.00 to 0.01, and therefore, of lesser importance.

**Table 4:** Properties of the workplace age-friendliness (WAF) scales (*N* = 1,725).

Variable	Core culture		Development		Health and well-being		Flexibility	
	*m*	*s*²	*m*	*s*²	*m*	*s*²	*m*	*s*²
**Total**	6.1	1.1	6.2	0.8	5.5	1.3	5.6	1.3
**Gender**	***	*R*² = 0.01	***	*R*² = 0.01	***	*R*² = 0.01	**	*R*² = 0.00
Male (*n* = 276)	5.9	1.10	6.0	0.9	5.3	1.4	5.4	1.3
Female (*n* = 1,407)	6.1	1.03	6.2	0.8	5.6	1.3	5.7	1.3
**Age groups** ^a^	*	*R*² = 0.00	*	*R*² = .00	***	*R*² = 0.01	***	*R*² = 0.01
Up to 44 years (*n* = 937)	6.2	0.9	6.2	0.8	5.6	1.2	5.7	1.1
45 years or older (*n* = 757)	6.0	1.2	6.2	0.9	5.4	1.5	5.5	1.4
**Education level**	**	*R*² = 0.01	***	*R*² = 0.01	***	*R*² = 0.01	***	*R*² = 0.01
Higher school certificate^b^ (*n* = 781)	6.2	1.0	6.3	0.8	5.7	1.3	5.7	1.2
No higher school certificate (*n* = 645)	6.0	1.1	6.1	0.8	5.4	1.4	5.5	1.3
**Organizations**	***	*R*² = 0.06	***	*R*² = 0.07	***	*R*² = 0.09	***	*R*² = 0.09
A (*n* = 68)	5.9	1.1	6.1	0.9	5.4	1.3	5.6	1.2
B (*n* = 86)	5.3	1.2	5.6	0.9	4.3	1.5	4.3	1.3
C (*n* = 268)	6.1	1.0	6.1	0.8	5.4	1.3	5.5	1.2
D (*n* = 331)	6.4	0.9	6.4	0.7	5.9	1.0	5.9	1.0
E (*n* = 748)	6.2	1.0	6.3	0.8	5.7	1.3	5.8	1.2
F (*n* = 224)	5.7	1.2	5.9	0.9	5.1	1.5	5.1	1.4
**Organization type**	***	*R*² = 0.03	***	*R*² = 0.04	***	*R*² = 0.03	***	*R*² = 0.03
Public (*n* = 224)	5.7	1.2	5.9	0.9	5.1	1.5	5.1	1.4
Profit (*n* = 331)	6.4	0.9	6.4	0.7	5.9	1.0	5.9	1.0
Nonprofit (*n* = 1,170)	6.1	1.0	6.2	0.8	5.5	1.3	5.60	1.3
**Industry**	***	*R*² = 0.05	***	*R*² = 0.05	***	*R*² = 0.06	***	*R*² = 0.06
Education (*n* = 154)	5.6	1.2	5.9	0.9	4.8	1.5	4.9	1.4
Health (*n* = 1,347)	6.2	1.0	6.3	0.8	5.7	1.2	5.8	1.2
Other (*n* = 224)	5.7	1.2	5.9	0.9	5.1	1.5	5.1	1.4
**Size**	***	*R*² = 0.01	***	*R*² = 0.01	***	*R*² = 0.01	***	*R*² = 0.02
50–250 employees (*n* = 68)	5.9	1.1	6.1	0.9	5.4	1.3	5.6	1.2
251–750 employees (*n* = 909)	6.0	1.1	6.1	0.9	5.4	1.4	5.5	1.3
751–1,500 employees (*n* = 748)	6.2	1.0	6.3	0.8	5.7	1.3	5.8	1.2

*Note*. WAF = workplace age-friendliness; *N* = 1,725; *n* valid cases, *m* mean, *s*^2^ standard deviation.

a Age groups were divided based on sample size per group.

b General qualification for university entrance; *R*^2^ coefficient of determination (ANOVA).

*** *p* ≤ .001.

** *p* ≤ .010.

* *p* ≤ .050.

## Discussion and implications

The aim of this study was to culturally adapt and validate the German version of the WAFM, particularly for use in the context of WHP within an aging workforce. Our goal was to empirically examine the psychometric properties of this instrument, demonstrating its reliability and validity. For this purpose, the responses of 1,725 employees across six Austrian organizations were analyzed rigorously, using advanced multivariate methods and SEM with latent variables. The results obtained are interpreted below, and their theoretical and practical implications, their limitations, and future research directions are discussed.

### Reliability and validity of the German WAFM

As proposed by previous studies (Eppler-Hattab et al., 2020a, 2024), the CFA of the German WAFM supported the four-factor solution and the differentiation of WAF by its dimensions of core culture, health and well-being, development, and flexibility. The proposed CFA model achieved acceptable to good fit, thus confirming the structural and construct validity of the German WAFM. In addition, its internal consistency and reliability also supported the use of the four-dimensional construct.

This study also tested the measurement equivalence of the WAFM dimensions for the first time. The restrictions introduced for (partial) metric and scalar measurement equivalence can be interpreted as such that the dimensions were measured invariantly across major socio-demographic groups (i.e., gender, age, and education). This result is essential, as invariant measurements are indispensable for a clear interpretation and ensure that the meaning of the instrument does not differ and can be considered as invariant quantities (Little, 2013).

The German WAFM also showed criterion (concurrent) validity across several conceptually related constructs. As expected, the WAFM scores correlated moderately to strongly with scales measuring organizational constructs, especially perceived organizational culture, appreciation and gratification on the part of managers and the organization, organizational climate, and organizational dissatisfaction. This examination supported our intention for the scales to measure the organizational perspective and not the individual only. Additionally, the validation process demonstrated that the WAFM scale scores differentiated more strongly between organizational aspects than across individual socio-demographic variables. These results not only indicate that the WAFM was able to detect relevant group differences but also highlight the prominence of the working conditions over individual differences for the concept under study.

### Theoretical implications

Validation of a scale is an ongoing process, involving continuous effort to gather evidence supporting its accuracy and reliability across different contexts and populations (Boateng et al., 2018). To our knowledge, this study is the first validating the WAFM in the specific context of health promotion initiatives. Previous validations were supported in Israeli organizations and in a more general context (Eppler-Hattab et al., 2020b, 2024). The validation of this construct in a variety of Austrian organizations provides further evidence of its psychometric properties in general and of its usefulness in the context of maintaining and promoting the health of an aging workforce over a prolonged working life in particular.

Furthermore, this study integrates the concepts of WAF and WHP in several important and meaningful ways. First, it highlights the centrality of primary prevention of workplace ill health through essential accommodative components such as work design, employment flexibility, and health-focused dimensions that are conceptually encompassed by WAF. This connection is a key concern that warrants further research in organizations employing aging and older workers, also to the advantage of the older workers themselves (Grosch & Scholl, 2020). Second, as organizations become more age diverse and multigenerational, a trend that is expected to continue to evolve (Low & Pok, 2025), human resource management literature addressing the health of aging and older workforces can benefit from the results of this study to pursue further age-related organizational research. For example, incorporating the WAFM in different cultures and languages (i.e., German, English, and Hebrew) can provide cross-country insights into cultural differences of WAF as well as into its application in global organizational environments.

Finally, and importantly, our study relates to theoretical and empirical evidence on organizational change management, suggesting that measuring organizational culture and climate is a necessary component of improving organizational effectiveness (Bartunek & Woodman, 2015; Schneider et al., 2013). This understanding is particularly salient for organizational change related to the health and well-being of an aging workforce, whose measurement can greatly contribute to maintaining organizational outcomes (Moen et al., 2017). In this regard, our validated construct can be utilized in organizational research addressing outcomes associated with the health components of WAF. As suggested by the criterion validity of the instrument demonstrated in our study, the WAFM may be related to organizational outcomes such as job satisfaction, job retention, and healthy culture, providing a basis for the development of process model research of organizational culture and climate in relation to employees’ healthy aging (for details and examples, see Boehm et al., 2021). Furthermore, potential benefits of a multigenerational workforce in German-speaking organizations, such as innovation-driving interaction, knowledge sharing and continuity, and operational resilience, although not explicitly enforced in the WAFM, can be captured and examined as interrelated with items from the core culture dimension (see Bobek et al., 2024).

### Practical implications

Alongside theoretical implications, the results also have practical implications. This is the first study to develop and validate a German-language version of the WAFM, which is now ready to use in German-speaking populations. This adds to existing instruments, and researchers as well as practitioners will be better equipped to assess the WAF of organizations and to accompany developments associated with age-inclusive and intergenerational organizational practice. For example, as the BMW study (Loch et al., 2010) exemplified with accommodative practices for retaining older workers, German-speaking organizations can use this instrument to address current demographic changes, as other ongoing European initiatives have demonstrated (e.g., Chand & Markova, 2019). Moreover, a health and well-being lens on the WAFM is a valuable extension of WHP measures. Similarly, WHP projects with a focus on older and aging workforces can use the WAFM as a baseline and situational analysis for developing environmentally directed and individual-directed measures.

Furthermore, the WAFM can be used to evaluate the implementation of supportive policies, practices, and procedures that demonstrate the ways in which a healthy age-friendly culture can be fostered and how such a climate can be manifested. According to the concept that based the WAFM (Eppler-Hattab et al., 2020b), these may include a combination of various measures at organizational or individual levels, such as development practices (e.g., lifelong learning processes, in which the knowledge, skills, and abilities of older workers are addressed and utilized), sustainment practices (e.g., the protection and promotion of health and well-being in the workplace, accompanied by training and qualification to update and upgrade professional skills and knowledge), modification practices (e.g., ways to improve performance and efficacy through job design and redesign that help adapt work demands to the evolving physical, cognitive, and social needs of older and aging workers), and flexibility practices (e.g., the development and provision of flexible employment arrangements for older workers who prefer or need to change their work-life balance). Regarding flexibility practices, it is worth noting that in Austria, for example, workplace flexibility is not inherently confidential information under labor law and may only be considered as confidential if it is not a common organizational practice (Kühteubl & Brandauer, 2022).

In the longer term, the WAFM dimensions might be used as a monitoring instrument to track developments in the culture and climate of organizations in relation to age and health as a system of health indicators within multigenerational management frameworks. In this sense, the WAFM can assist German-speaking organizations in addressing demographic challenges by improving the work ability and employability of their aging workforce in relation to diverse health aspects. It can also contribute to more equitable organizations by reducing the vulnerability and (health) inequality of individuals in the late stages of their working lives and during critical career-life events, such as the transition to retirement or post-employment life. By focusing on organizational and cultural change, this approach has the potential for cross-generational impact, for example, by ensuring that the transition to retirement is perceived as a positive event for both the organization and the individual.

### Limitations and future research directions

The results of this study should be interpreted in relation to some limitations, which should be considered in future applications of the German WAFM. First, as it addresses age-diverse organizations, the use of the WAFM is advised for organizations with a certain number of older employees; it is recommended for use in German-speaking organizations with a minimum of 10% of employees aged 55 and above for large organizations and 15% for medium-sized organizations (Eppler-Hattab et al., 2024) but, as argued above, it should be administered to employees of all ages.

Second, the current study results are based on online organizational surveys. For an additional foundation, construct validity could be also demonstrated by alternative survey methods, such as, for example, face-to-face or telephone interviewing techniques. Future applications should, therefore, test the validity of the WAFM by applying multitrait-multimethod studies, which could also shed light on method-specific effects that may potentially influence the measurement (Helm, 2022). Further analysis techniques (e.g., classification methods) might also be helpful in strengthening the discriminant validity assessment of our scale. We also recommend that future studies use data on variables relevant to the health of an aging workforce, such as the frequency of health screenings or disparities in the health status of diverse populations, such as migrant domestic workers, or aging LGBTQ workers (Eppler-Hattab & Steindórsdóttir, 2025).

Third, the current validation was based on a wide sample size but was limited to certain Austrian organizations, sectors and industries, and should, therefore, be placed on a broader basis for further generalizability (e.g., industry-specific benchmarks) in future studies. Although this study demonstrated that perceptions of WAF vary between organizational contexts, factorial validity should also be tested in a multilevel research design using an appropriate sample of organizations (e.g., in multilevel-organizational analysis with at least 30 organizations, after reducing degrees of freedom associated with model variables; Hox et al., 2017). Against the conventional assumption of CFA, multilevel CFA does not need to assume that data are independent from the organizational context (e.g., across diverse age-friendly cultures, practices, and climates), and this would explicitly test the relationship between latent WAF dimensions and their items across organizations (Hox et al., 2017).

Finally, for the purpose of validating the German WAFM in this study, the data used were cross-sectional within organizations. To test intervention-based changes across time, it is also necessary to demonstrate the (test–retest) reliability and validity of the measurement quality over several measurement occasions, for example, by using a pretest–post-test design. The existence of longitudinal measurement equivalence can ensure that the WAFM indeed measures what it is supposed to measure, regardless of when it is performed. This test is crucial because the relationship between the instrument and the underlying latent variables can change over time, such as when an intervention lies between the measurement opportunities (e.g., due to age-related WHP actions taken). The existence of longitudinal factorial measurement invariance is, therefore, fundamental to the conclusion that observed changes over the observation period can be attributed to changes in the latent dimensions of the scale and not to its characteristics (see Widaman & Olivera-Aguilar, 2023).

## Conclusion

In this article, we developed and validated a German-language version of the WAFM, considering the specific context of its use in promoting the health of an aging workforce. We believe that the German-language WAFM and its demonstrated psychometric properties as introduced in this study have the potential to advance the workplace health of an aging and older workforce in a wide range of organizations in relevant countries. We hope that both professionals and researchers will benefit from its results and implications in a way that will contribute both to organizations employing an older and age-diverse workforce and to the healthy and constructive employment of older workers themselves.

## Data Availability

The German-language version of the WAFM is available for use by researchers and practitioners. The data collected in the current study cannot be shared publicly due to confidentiality requirements and agreements.
